# Association of *FOXF2* gene polymorphisms with ischemic stroke in Chinese Han population

**DOI:** 10.18632/oncotarget.21263

**Published:** 2017-09-23

**Authors:** Chang-He Shi, Mi-Bo Tang, Shao-Hua Li, Zhi-Jie Wang, Xin-Jing Liu, Lu Zhao, Yuan Gao, Yu-Sheng Li, Shi-Lei Sun, Jun Wu, Bo Song, Yu-Ming Xu

**Affiliations:** ^1^ Department of Neurology, The First Affiliated Hospital of Zhengzhou University, Zhengzhou University, Zhengzhou 450000, Henan, China

**Keywords:** FOXF2, single nucleotide polymorphism, ischemic stroke, large artery atherosclerotic stroke, small vessel disease stroke

## Abstract

Recently, a novel locus at chromosome 6p25 (rs12204590, near *FOXF2*) associated with an increased risk of stroke in European populations was identified. However, whether polymorphisms in *FOXF2* are also associated with the incidence of ischemic stroke in other populations remains unknown. In this case-control study, 803 Chinese Han patients with ischemic stroke and 803 matched control individuals were enrolled. Four tag SNPs and rs12204590 located in or near *FOXF2* were selected, and the associations between genotypes/alleles and ischemic stroke were analyzed. In our study, we did not detect an association between the previously reported locus rs12204590 and ischemic stroke. By the genotype analysis, a novel SNP rs1711972, near *FOXF2*, was observed to be associated with an increased risk of ischemic stroke(CA genotype, adjusted OR = 1.35; 95% CI, 1.07 to 1.70), but not significantly after Bonferroni corrections for multiple tests. However, in the subgroup analysis, we discovered that rs1711972 was associated with an increased risk of large-artery atherosclerotic stroke in the additive model (*P* = 0.020; CA genotype, adjusted OR = 1.50; 95%CI, 1.09 to 2.07) and dominant model (*P* = 0.010; OR = 1.47; 95%CI, 1.09 to 1.99). Collectively, these results indicate that a novel SNP near *FOXF2* may influence the risk of large-artery atherosclerotic stroke in Chinese Han population.

## INTRODUCTION

Stroke is the leading neurological cause of death and long-term disability worldwide [1, 2]. In China, millions of people suffer from stroke, and there are approximately 2.5 million new cases of patients with stroke and 7.5 million stroke survivors each year [3, 4]. Although the various risk factors underlying this critical pathological condition have been identified in different geographic regions [5, 6], the genetic background has been proposed as a possible cause and promoting factor for the occurrence of irregularity in the risk of stroke among ethnic populations [7]. Therefore, the other genetic loci that are associated with the susceptibility to stroke should be identified in these populations.

Risk loci associated with stroke and its subtypes have been identified in several genes [8, 9], such as *PITX2*, *ZFHX3*, *HDAC9*, *NINJ2*, *ABO*, *APOE* and *PMF1* [3, 10–17]. Recently, a novel locus at chromosome 6p25 (rs12204590, near *FOXF2*) has been observed to be associated with an increased risk of stroke in European populations [18]. The *FOXF2* gene is located in chromosome 6p25.3. It has been revealed that chromosome 6p25.3 is associated with an increase in the appearance of white matter hyperintensities in the general population [18]. FOXF2 is first expressed in the neural crest cells, and in mice, it regulates the pathways that are involved in the differentiation of mural cells (pericytes and vascular smooth muscle cells), including the PDGF-β and serum response factor pathways [19]. FOXF2 is also required for the development of the blood-brain barrier [20]. The conditional deletion of FOXF2 in adult mice led to the development of cerebral infarction, reactive gliosis, and cerebral microhemorrhage [18]. Patients with rare segmental deletions of FOXF2 also exhibited an increased appearance of white matter hyperintensities. As large-artery atherosclerotic (LAA) stroke and small-vessel disease (SVD)-related stroke are the most pervasive subtypes in the Chinese Han population [21], and increasing evidence has revealed that the genetic risks vary depending on the subtypes of ischemic stroke [22], it is important to determine whether the variants in or near *FOXF2* are associated with the increased risk of ischemic stroke and its subtypes.

To investigate the possible role of rs12204590 and other new loci in or near *FOXF2* that are associated with ischemic stroke, a case-control study of 803 cases and 803 controls was performed.

## RESULTS

### Clinical characteristics

As shown in Table 1, there was no significant difference in gender (*P* = 0.166) and age (*P* = 0.057) between the patients and controls. The results for the prevalence of hyperlipidemia (*P* = 0.583) and smoking (*P* = 0.124) were also similar. Conversely, there was a significant deviation with respect to the history of hypertension (*P* < 0.01) and diabetes mellitus (DM) (*P* < 0.01) revealed between the patient and control groups. In the subsequent analyses, therefore these confounding factors (age, sex, hypertension, DM, hyperlipidemia, and smoking) were adjusted for to estimate the effect of these SNPs on the susceptibility to ischemic stroke.

**Table 1:** Characteristics of cases and controls

Variables	Cases(*n* = 803)	Controls(*n*= 803)	*P* value
Age(years)			0.057
≥ 60	393	355	
< 60	410	448	
Sex			0.166
Male	555	529	
Female	248	274	
Hypertension			< 0.001
No	276	597	
Yes	527	206	
Diabetes			< 0.001
No	633	768	
Yes	170	35	
Hyperlipidemia			0.583
No	383	394	
Yes	420	409	
Smoking			0.124
No	555	583	
Yes	248	220	

### Association of SNP with ischemic stroke

All the selected SNPs were in Hardy–Weinberg equilibrium (*P* > 0.05) in the control group (Table 2). The univariate analysis indicated that there were significant differences in allelic frequencies of the rs1711972 A > C polymorphism between the groups (*P* = 0.014; OR = 1.22; 95% CI, 1.04 to 1.42). The dominant-effect model also indicated that the rs1711972 locus was associated with an increased risk of ischemic stroke (CC/CA vs. AA, *P* = 0.011; OR = 1.34; 95% CI, 1.07 to 1.67). The frequencies of the AA, CA and CC genotypes in the patients were 50.44%, 41.72%, and 7.85% respectively, and in the controls, their frequencies were 56.78%, 36.81%, and 6.41% respectively (Table 3). The CA genotype of rs1711972 remained associated with an increased risk of ischemic stroke in the additive model (OR = 1.35; 95% CI, 1.07 to 1.70); However, there was no significant association after the correction for multiple tests (*P*^corr^ = 0.019). According to the Hosmer-Leme show goodness-of-fit test, this model was well calibrated (*P* = 0.700 for rs1711972). The other four selected SNPs showed no detectable association with ischemic stroke in this study.

**Table 2:** Information of selected SNPs of FOXF2 gene region in a Chinese population

SNP	Chr	Location on Chr	Allele	MAF(CHB)^a^	*P* value^b^
rs12204590	6	5081426	T:A	0.010	1.000
rs1711972	6	6240084	A:C	0.244	0.958
rs41300825	6	1391620	G:C	0.102	0.764
rs732835	6	1393000	G:C	0.311	0.989
rs910023	6	1394185	G:A	0.291	0.999

SNP single nucleotide polymorphism, Chr chromosome, MAF minor allele frequency.

^a^ From 1000 Genomes Project database.

^b^ *P* value Hardy-Weinberg equilibrium in control.

**Table 3:** Association between tag SNPs and risk of ischemic stroke

Genotypes		Cases *n* = 803	Controls *n* = 803	*P* value	*P^corr^*	Crude OR (95%CI)	Adjusted OR^a^ (95%CI)
rs12204590							
	TT	786	791	0.351		1.00	1.00
	AT	17	12			1.43 (0.68–3.00)	1.20 (0.52–2.79)
	AA	0	0				
	T	1589	1594	0.353		1.00	—
	A	17	12			1.70 (0.34–1.48)	—
rs1711972							
	AA	405	456	0.037	1.000	1.00	1.00
	CA	335	295		0.019	1.28 (1.04–1.57)	1.35 (1.07–1.70)
	CC	63	52		0.118	1.36 (0.92–2.01)	1.32 (0.85–2.05)
	A	1145	1207	0.014		1.00	—
	C	461	399			1.22 (1.04–1.42)	—
	CC+CA	398	347	0.011		1.29 (1.06–1.57)	1.34 (1.07–1.67)
rs41300825							
	GG	642	614	0.235		1.00	1.00
	CG	154	180			0.82 (0.64–1.04)	0.82 (0.63–1.08)
	CC	7	9			0.74 (0.28–2.01)	0.73 (0.24–2.20)
	G	1438	1408	0.096		1.00	—
	C	168	198			0.83 (0.67–1.03)	—
	CC+CG	161	189	0.091		0.82 (0.64–1.03)	0.82 (0.63–1.07)
rs732835							
	GG	418	419	0.915		1.00	1.00
	CG	329	324			1.02 (0.83–1.25)	1.07 (0.85–1.35)
	CC	56	60			0.94 (0.63–1.38)	1.01 (0.65–1.57)
	G	1165	1162	0.906		1.00	—
	C	441	444			0.99 (0.85–1.16)	—
	CC+CG	385	384	0.960		1.01 (0.83–1.22)	1.06 (0.85–1.32)
rs910023							
	GG	447	451	0.980		1.00	1.00
	GA	305	302			1.02 (0.83–1.25)	1.07 (0.85–1.35)
	AA	51	50			1.03 (0.68–1.56)	1.15 (0.73–1.84)
	G	1199	1204	0.839		1.00	—
	A	407	402			1.02 (0.87–1.20)	—
	AA+GA	356	352	0.841		1.02 (0.84–1.24)	1.08 (0.87–1.35)

*CI* confidence interval, *OR* odds ratio.

^a^ Adjusted for age, sex, hypertension, diabetes mellitus, hyperlipidemia, and smoking.

*P*^corr^ corrected *P* value by Bonferroni correction.

### Subgroup analysis

To explore the effect of SNPs on the risk of the subtypes of stroke, the subjects were stratified by the TOAST subtypes. As shown in Table 4, the univariate analyses revealed significant deviations in the genotype frequencies of rs1711972 between the patients with LAA stroke and the healthy controls (*P* = 0.020). Multivariate logistic regression analysis, which was adjusted for the aforementioned confounding factors, demonstrated that the CA genotype of rs1711972 remained associated with the increased risk of LAA stroke (OR = 1.50; 95% CI, 1.09 to 2.07). The frequencies of the AA, CA, and CC genotypes were 47.67%, 42.79%, and 9.54% respectively, in the patients, and 57.21%, 34.88%, and 7.91%, respectively, in the controls. A significant association with the increased risk of LAA stroke remained for the CA genotype after the correction for multiple tests (*P*^corr^ = 0.008). *P* = 0.010 for the allelic distribution of rs 1711972 and *P* = 0.033 for that of rs41300825 in association with LAA stroke. The dominant-effect model revealed that the rs1711972 locus (CC/CA vs. AA, *P* = 0.010; OR = 1.47; 95% CI, 1.09 to 1.99) was associated with the increased risk of LAA stroke, and the rs41300825 locus (CC/CG vs. GG, *P* = 0.038; OR = 0.68; 95% CI, 0.47 to 0.98) was associated with a decreased risk of LAA stroke. The results of the Hosmer-Leme show goodness-of-fit test were *P* = 0.176 for rs1711972, and *P* = 0.105 for rs41300825. As shown in Table 5, no association between the involved SNPs and the risk of SVD stroke was detected.

**Table 4:** Association between tag SNPs and risk of LAA stroke

Genotypes		Cases *n* = 430	Controls *n* = 430	*P* value	*P^corr^*	Crude OR (95%CI)	Adjusted OR^a^ (95%CI)
rs12204590							
	TT	422	424	0.591		1.00	1.00
	AT	8	6			1.34 (0.46–3.89)	0.87 (0.27–2.78)
	AA	0	0				
	T	852	854	0.593		1.00	—
	A	8	6			1.37 (0.46–3.87)	—
rs1711972							
	AA	205	246	0.020	1.000	1.00	1.00
	CA	184	150		0.008	1.47 (1.11–1.96)	1.50 (1.09–2.07)
	CC	41	34		0.139	1.45 (0.89–2.36)	1.36 (0.78–2.37)
	A	594	642	0.010		1.00	—
	C	266	218			1.34 (1.07–1.62)	—
	CC+CA	225	184	0.010		1.47 (1.21–1.92)	1.47 (1.09–1.99)
rs41300825							
	GG	350	325	0.099		1.00	1.00
	CG	77	99			0.72 (0.52–1.01)	0.70 (0.48–1.02)
	CC	3	6			0.46 (0.12–1.88)	0.42 (0.10–1.83)
	G	777	749	0.033		1.00	—
	C	83	111			0.72 (0.53–0.98)	—
	CC+CG	80	105	0.038		0.71 (0.51–0.98)	0.68 (0.47–0.98)
rs732835							
	GG	236	220	0.494		1.00	1.00
	CG	165	182			0.85 (0.64–1.12)	0.91 (0.67–1.24)
	CC	29	28			0.97 (0.56–1.68)	1.21 (0.65–2.24)
	G	637	622	0.414		1.00	—
	C	223	238			0.92 (0.74–1.13)	—
	CC+CG	194	210	0.520		0.87 (0.66–1.13)	0.94 (0.70–1.27)
rs910023							
	GG	248	238	0.568		1.00	1.00
	GA	155	169			0.89 (0.66–1.17)	0.95 (0.70–1.31)
	AA	27	23			1.13 (0.63–2.02)	1.44 (0.75–2.78)
	G	651	645	0.758		1.00	—
	A	209	215			0.97 (0.78–1.20)	—
	AA+GA	182	192	0.492		0.91 (0.70–1.19)	1.01 (0.74–1.36)

*CI* confidence interval, *OR* odds ratio.

^a^ Adjusted for age, sex, hypertension, diabetes mellitus, hyperlipidemia, and smoking.

*P*^corr^ corrected *P* value by Bonferroni correction.

**Table 5:** Association between tag SNPs and risk of SVD stroke

Genotypes		Cases *n* = 373	Controls *n* = 373	*P* value	Crude OR (95%CI)	Adjusted OR^a^ (95%CI)
rs12204590						
	TT	364	367	0.437	1.00	1.00
	AT	9	6		1.51 (0.53–4.29)	1.27 (0.39–4.09)
	AA	0	0			
	T	737	740	0.178	1.00	—
	A	9	6		2.25 (0.69–7.35)	—
rs1711972						
	AA	200	211	0.231	1.00	1.00
	CA	151	132		1.22 (0.90–1.65)	1.32 (0.95–1.85)
	CC	22	30		0.77 (0.43–1.39)	0.92 (0.48–1.75)
	A	551	554	0.859	1.00	—
	C	195	192		1.02 (0.81–1.29)	—
	CC+CA	173	162	0.418	1.13 (0.84–1.50)	1.25 (0.91–1.72)
rs41300825						
	GG	292	279	0.688	1.00	1.00
	CG	77	88		0.89 (0.63–1.26)	0.81 (0.55–1.19)
	CC	4	6		0.68 (0.19–2.43)	0.67 (0.16–2.77)
	G	661	646	0.239	1.00	—
	C	85	100		0.83 (0.61–1.13)	—
	CC+CG	81	94	0.262	0.82 (0.59–1.16)	0.80 (0.55–1.16)
rs732835						
	GG	182	192	0.705	1.00	1.00
	CG	164	158		1.10 (0.81–1.48)	1.10 (0.79–1.54)
	CC	27	23		1.24 (0.69–2.24)	1.45 (0.75–2.79)
	G	528	542	0.421	1.00	—
	C	218	204		1.10 (0.88–1.40)	—
	CC+CG	191	181	0.464	1.13 (0.84–1.48)	1.14 (0.83–1.57)
rs910023						
	GG	199	205	0.715	1.00	1.00
	GA	150	149		1.04 (0.77–1.40)	1.05 (0.76–1.46)
	AA	24	19		1.30 (0.69–2.45)	1,48 (0.73–2.99)
	G	548	559	0.515	1.00	—
	A	198	187		1.08 (0.86–1.36)	—
	AA+GA	174	168	0.659	1.07 (0.80–1.42)	1.10 (0.80–1.51)

*CI* confidence interval, *OR* odds ratio.

^a^ Adjusted for age, sex, hypertension, diabetes mellitus, hyperlipidemia, and smoking.

### Haplotype and LD analyses

The LD analysis revealed a block of LD, including three SNPs, rs41300825, rs732835, and rs910023 (R^2^ = 0.831) (Supplementary Figure 1). In the haplotype analysis, after adjusting for age, sex, hypertension, DM, hyperlipidemia and smoking, none of the Haplotypes were observed to be associated with stroke (Table 6).

**Table 6:** Hyplotype analysis between cases and controls

HapMap block	Hyplotype^a^	Case (*n*, frequencies)	Control (*n*, frequencies)	Adjusted OR (95%CI)^b^	*P* value^b^
Block1					
rs41300825, rs732835,rs910023	CGG	170, 0.105	195, 0.121	0.86 (0.69–1.07)	0.173
rs41300825, rs732835,rs910023	GCG	47, 0.029	42, 0.026	1.13 (0.74–1.72)	0.583
rs41300825, rs732835,rs910023	GGG	987, 0.612	965, 0.597	1.07 (0.93–1.23)	0.379
rs41300825, rs732835,rs910023	GCA	396, 0.246	400, 0.248	0.99 (0.84–1.16)	0.902

*CI* confidence interval, *OR* odds ratio

^a^ Haplotypes with frequency less than 1 % were omitted

^b^Adjusted for age, sex, hypertension, diabetes mellitus, hyperlipidemia, and smoking.

### MDR for SNP–SNP interactions

The effect of the interaction between the selected SNPs on the risk of ischemic stroke was analyzed by MDR. As shown in Table 7, in the single-locus model, rs1711972 was the most probable contributing factor to susceptibility to ischemic stroke (testing accuracy = 0.5318, CVC = 10/10, *P* = 0.420); However, the results were not significant.

**Table 7:** SNP-SNP interactions analyzed with MDR

Model	Bal.Acc.Cvtraining	Bal.Acc.Cv testing	CVC	*P* value^a^
rs1711972	0.5318	0.5318	10/10	0.420
rs1711972, rs41300825	0.5434	0.5255	7/10	0.513
rs1711972, rs41300825, rs910023	0.5515	0.5162	6/10	0.682
rs1711972, rs41300825, rs732835, rs910023	0.5561	0.5118	6/10	0.764

*CVC* cross-validation consistency.

^a^*P* value based on 1000 permutations.

## DISCUSSION

In this study, we identified a novel SNP, rs1711972, which was associated with the susceptibility to LAA stroke using the additive model, dominant model, and allelic analyses. This is the first study to investigate the relationship between the SNPs in *FOXF2* and ischemic stroke in Chinese Han population.

*FOXF2* is located on chromosome 6p25.3, and consists of two exons and one intron spanning approximately 5–6 kb [18]. This gene encodes a forkhead box transcription factor, which is involved in the regulation of various developmental and biological processes [23, 24]. Previous studies revealed that *FOXF2* is expressed in neural crest cells, which are progenitors of cerebrovascular mural cells. Patients with segmental deletions of this gene showed the extensive, confluent appearance of white matter hyperintensities, and animal experiments showed that conditional *FOXF2* mutations induced the development of cerebral infarction, microhemorrhage, and defects in the differentiation of cerebral vascular mural cells [20, 25]; Recently, a common variant, rs12204590 near *FOXF2,* was found to be associated with increased susceptibility to stroke. The rs1711972 locus is located in the region between *FOXQ1* and *FOXF2*, which contains enhancers and DNaseI hypersensitive regions. For this reason, we speculate that rs1711972 may regulate *FOXF2* expression. However, the significant association of rs12204590 with susceptibility to SVD stroke in Chinese Han population was not observed, which appears to conflict with the reports of previous studies on European populations. The discrepancy may be attributed to genetic heterogeneity due to geographic or ethnic distribution and minor variations in the allele frequencies of *FOXF2* between Chinese and European populations (rs12204590, frequency of A allele in Europeans = 0.1899, frequency of A allele in East Asians = 0.0030). The relationship between SNP-SNP interactions and the risk of ischemic stroke was also analyzed by MDR. However, no significant interaction among these five SNPs was observed.

Our study had several limitations. First, there might be potential selection bias owing to the hospital-based study design. Second, the sample size was not particularly large, and certain positive results might not have been detected. Furthermore, as there are many factors that have been revealed to be associated with stroke, the biological mechanisms of the association between polymorphisms and susceptibility to ischemic stroke are not clarified yet, which requires additional *in vitro* and *in vivo* studies in the future.

In summary, we identified a novel *FOXF2* SNP (rs1711972), which may be used as a candidate biomarker of ischemic stroke and LAA stroke in the Chinese Han population. Simultaneously, the early detection of SNPs would greatly contribute to the prevention of ischemic stroke, particularly LAA stroke. Future studies on different population groups and well-designed functional experiments are necessary to verify and extend our findings.

## MATERIALS AND METHODS

### Ethics statement

This study was approved by the Ethics Review Board of The First Affiliated Hospital of Zhengzhou University (Zhengzhou, China). All the enrolled patients provided their written informed consent to participate.

### Study subjects

The study enrolled 803 patients with ischemic stroke and 803 healthy controls. The patients were selected from the hospitalized patients who experienced their first-ever stroke between July 2011 and March 2016. All the patients were initially assessed for the eligibility to participate. The inclusion criteria for the patients were obtained from certain published papers and guides as follows [3, 4]: (1) Chinese Han ethnicity, (2) age of 18 years or above, (3) incidence of first-ever ischemic stroke diagnosed within 14 days. The exclusion criteria were: (1) incidence of severe heart, lung, liver, and kidney dysfunction, (2) incidence of malignancies, (3) incidence of hematologic diseases, (4) incidence of autoimmune, inflammatory, or systematic diseases. The stroke-free control subjects were selected from local residents who underwent physical examinations in the sample hospital, and their inclusion criteria were as follows: (1) Chinese Han ethnicity, (2) age of 18 years or above, (3) no history of atherosclerotic, cardiovascular, and cerebrovascular diseases; (4) regular physical examinations.

### Selection of SNPs

The acquisition of SNP data was based on the HapMap database, and tag SNPs were determined using the Haploview Software version 4.2(http://www.broadinstitute.org/haploview/haploview) [26], and selected using the following filters: (1) r^2^ = 0.8, (2) minor allele frequency (MAF) ≥ 10 %, (3) Hardy-Weinberg equilibrium test *P* value ≥ 0.05. Finally, a total of 5 SNPs were selected for further investigation: Four were novel tag SNPs (rs1711972, rs41300825, rs732835, and rs910023), and one was the recently reported risk locus for stroke (rs12204590). Table 2 shows the chromosomal location and information on the population distribution of these SNPs. Linkage disequilibrium (LD) between the SNPs was calculated using the Haploview Software version 4.2, and is included in Supplementary Figure 1.

### DNA isolation and genotyping

DNA was isolated as described previously [4]. Five milliliters (ml) of peripheral blood was collected from each subject in an vacutainer tube with EDTA. Genomic DNA was extracted using a DNA isolation kit, according to the manufacturer's instructions. Genotyping was performed by the improved multiple ligation-detection reaction (iMLDR), with technical support from the Center for Human Genetics Research. For quality control and validation purposes, genotyping was repeated on 10% of the samples, and the concordance rate for the replicate samples was 100%. The sequences of the primer pairs used are shown in the Supplementary Table 1.

### SNP–SNP interactions

The SNP-SNP interactions were analyzed as described previously [3]. Multifactor dimensionality reduction (MDR) (version 3.0_0_2) and MDR-permutation testing ( MDRpt, version 1.0_beta_2) were adopted to evaluate the effect of the SNP-SNP interactions on the risk of developing ischemic stroke [27, 28]. Cross-validation and permutation tests were conducted to evaluate the validity of these models for predicting the risk of ischemic stroke. For multi loci models, the highest level of testing accuracy and the cross-validation consistency (CVC) were used for determining the best candidate interaction model. Furthermore, a testing accuracy level greater than 0.5 was required in the true-positive models. Statistical significance was analyzed by the implementation of 1000-fold permutation testing methods, and a *P* value less than 0.05 was considered statistically significant.

### Statistical analysis

A chi-square goodness of fit test was conducted to determine the Hardy-Weinberg equilibrium of the frequency distribution of each SNP in the control group (Table 2). Chi-square tests were also conducted to compare the differences in categorical covariates between the patients and controls. Logistic regression analysis was performed to evaluate the association of each SNP with the risk of ischemic stroke and its subtypes. The Hosmer-Leme show goodness of fit test was performed to assess the calibration of the logistic regression model. For single comparison, a *P* value < 0.05 was considered statistically significant. For multiple comparisons, the Bonferroni correction was adopted for correcting the *P* values. A *P* value < 0.05/number of comparisons was considered statistically significant. The analysis of linkage disequilibrium (LD) was performed, and haplotype blocks were identified using Haploview v4.2, then the haplotypes were reconstructed using the PHASE software v2.1;the effect of the potential interactions between these five tSNPs on susceptibility to ischemic stroke was analyzed by MDR. The statistical analyses were conducted using IBM SPSS Statistics version 22.0 (Armonk, NY, USA: IBM Corp.).

## SUPPLEMENTARY MATERIALS FIGURES AND TABLES



## Abbreviations

LAA: Large artery atherosclerotic
SVD: Small vessel disease
CVC: Cross-validation consistency
MAF: Minor allele frequency
LD: Linkage disequilibrium
