# Supplementary material for: Association of FOXF2 gene polymorphisms with ischemic stroke in Chinese Han population
Source: Oncotarget. 2017 Sep 23;8(52):89867–75. doi: 10.18632/oncotarget.21263 (PMC5685715; doi:10.18632/oncotarget.21263)
Supplement: Supplementary file 1 [file oncotarget-08-89867-s001.pdf]

Association of *FOXF2* gene polymorphisms with ischemic stroke in Chinese Han population

SUPPLEMENTARY MATERIALS

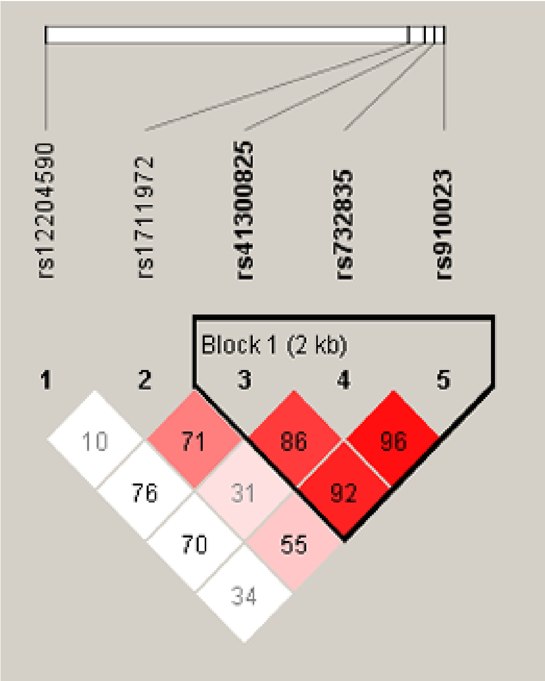

Supplementary Figure 1: Linkage disequilibrium (LD) plot for five SNPs of FOXF2 gene in 803 ethnic Chinese Han controls. This plot was generated by the Haploview program. A block was determined. The rs number corresponds to the SNP name. D' indicates the degree of LD between the two SNPs.

Supplementary Table 1: Primers used in Polymerase Chain Reaction to repeat genotyping on 10% of the samples

Gene	Primers	Sequences
rs12204590	Forward	TGTAACCCAAAGGTGCCAGT
	Reverse	TGAACAAAACAGGAAGAAATCC
rs1711972	Forward	AAGAAGTGGAAGCAAATGCAA
	Reverse	AGGGCCGATACTGACTCCAT
rs41300825	Forward	TAGGAGGTCTGAGGGCACTG
	Reverse	TCGGGACAGAAGTACAGCAA
rs732835	Forward	GCTTAGCAGCCTTGGAGAAA
	Reverse	CAAGACAGCGAGGAAGGAAG
rs910023	Forward	CGGGCGAATGGACCTAAC
	Reverse	AAAAGCACGCCTCTTCTCCT
